# Relationships between structure, process and outcome to assess quality of integrated chronic disease management in a rural South African setting: applying a structural equation model

**DOI:** 10.1186/s12913-017-2177-4

**Published:** 2017-03-23

**Authors:** Soter Ameh, Francesc Xavier Gómez-Olivé, Kathleen Kahn, Stephen M. Tollman, Kerstin Klipstein-Grobusch

**Affiliations:** 1 Medical Research Council/Wits University Rural Public Health and Health Transitions Research Unit (Agincourt), School of Public Health, Faculty of Health Sciences, University of the Witwatersrand, Johannesburg, South Africa; 2 Department of Community Medicine, Faculty of Medicine, College of Medical Sciences, University of Calabar, Calabar, Cross River State Nigeria; 3 The International Network for the Demographic Evaluation of Populations and Their Health in Developing Countries (INDEPTH), Accra, Ghana; 4 Umeå Centre for Global Health Research, Epidemiology and Global Health, Umeå University, Umeå, Sweden; 5 Division of Epidemiology and Biostatistics, School of Public Health, University of the Witwatersrand, Johannesburg, South Africa; 6 Julius Global Health, Julius Center for Health Sciences and Primary Care, University Medical Center Utrecht, Utrecht, The Netherlands

**Keywords:** Integrated Chronic Disease Management (ICDM) Model, Avedis donabedian, Constructs, Quality of care, Satisfaction, Chronic communicable diseases, Non-communicable chronic diseases, Structural equation model, Primary Health Care (PHC), Mpumalanga province, South Africa

## Abstract

**Background:**

South Africa faces a complex dual burden of chronic communicable and non-communicable diseases (NCDs). In response, the Integrated Chronic Disease Management (ICDM) model was initiated in primary health care (PHC) facilities in 2011 to leverage the HIV/ART programme to scale-up services for NCDs, achieve optimal patient health outcomes and improve the quality of medical care. However, little is known about the quality of care in the ICDM model. The objectives of this study were to: i) assess patients’ and operational managers’ satisfaction with the dimensions of ICDM services; and ii) evaluate the quality of care in the ICDM model using Avedis Donabedian’s theory of relationships between structure (resources), process (clinical activities) and outcome (desired result of healthcare) constructs as a measure of quality of care.

**Methods:**

A cross-sectional study was conducted in 2013 in seven PHC facilities in the Bushbuckridge municipality of Mpumalanga Province, north-east South Africa - an area underpinned by a robust Health and Demographic Surveillance System (HDSS). The patient satisfaction questionnaire (PSQ-18), with measures reflecting structure/process/outcome (SPO) constructs, was adapted and administered to 435 chronic disease patients and the operational managers of all seven PHC facilities. The adapted questionnaire contained 17 dimensions of care, including eight dimensions identified as priority areas in the ICDM model - critical drugs, equipment, referral, defaulter tracing, prepacking of medicines, clinic appointments, waiting time, and coherence. A structural equation model was fit to operationalise Donabedian’s theory, using unidirectional, mediation, and reciprocal pathways.

**Results:**

The mediation pathway showed that the relationships between structure, process and outcome represented quality systems in the ICDM model. Structure correlated with process (0.40) and outcome (0.75). Given structure, process correlated with outcome (0.88). Of the 17 dimensions of care in the ICDM model, three structure (equipment, critical drugs, accessibility), three process (professionalism, friendliness and attendance to patients) and three outcome (competence, confidence and coherence) dimensions reflected their intended constructs.

**Conclusion:**

Of the priority dimensions, referrals, defaulter tracing, prepacking of medicines, appointments, and patient waiting time did not reflect their intended constructs. Donabedian’s theoretical framework can be used to provide evidence of quality systems in the ICDM model.

**Electronic supplementary material:**

The online version of this article (doi:10.1186/s12913-017-2177-4) contains supplementary material, which is available to authorized users.

## Background

South Africa faces a complex dual burden of chronic communicable (HIV and TB) and chronic non-communicable diseases (NCDs - e.g. cardiovascular diseases, diabetes, cancer and chronic respiratory diseases), with the prevalence of HIV estimated at 10% in 2014 [1] and mortality due to NCDs estimated at 43% in 2012 [2]. Effectively responding to this dual burden of chronic diseases requires an integrated approach to the delivery of care at the primary health care (PHC) level.

The Joint United Nations Programme on HIV/AIDS (UNAIDS) recommends a globally comprehensive and integrated approach to the delivery of chronic disease care. This approach requires leveraging HIV programmes to support or scale-up services for NCDs [3, 4]. There is evidence that the integrated management of chronic diseases leads to improvement in patient health outcomes (e.g., CD4 count, glycosylated haemoglobin, and blood pressure) and patient satisfaction with the delivery of chronic disease care [5]. Beyond the UNAIDS mandate for the implementation of an integrated chronic care model, integrating services for HIV and NCDs could also minimise fragmented chronic disease care arising from the management of the HIV pogramme in a ‘silo’ within the general healthcare system, leverage resources and more efficiently meet patients’ healthcare needs [6–8].

In response to UNAIDS recommendation to integrate HIV and NCD services, the National Department of Health (NDoH) in South Africa initiated the Integrated Chronic Disease Management (ICDM) model [9]. The pilot of the ICDM model commenced in 2011 in selected PHC facilities in three of South Africa’s nine provinces (Gauteng, Mpumalanga and North West), [9] with the expectation of enhancing the quality of chronic disease services and improving patient health outcomes.

At the crux of the ICDM operational framework are facility reorganisation to improve operational efficiency and quality of care in the health facilities; “assisted” self-management to promote individual responsibility in the communities; and health promotion and population screening in the population [9]. The facility component entails many areas of focus such as: designation of chronic care area; use of guidelines for management of chronic diseases; human resource audit; capacity building; supply of critical medicines; prepacking of medication; and appropriate referral. To prepare the community for chronic disease care, each clinic has a PHC outreach team operating within the community that the clinic serves, and consists of one professional nurse, three staff nurses, and six Community Health Workers (CHWs). With the outreach team responsible for 6000 individuals in 1500 households (250 households per 1 CHW), it is anticipated that at least 80% of defined health problems of the catchment population would be managed [9]. This study focuses on the facility component of the ICDM model.

Multiple meanings of “Integrated health care” exist in the literature. These include the provision of health care for multiple diseases at one service delivery point (e.g. integrated management of childhood illness); continuity of care over time across different levels of health care (e.g. an appropriate referral system); integrating vertical programmes (programmes that are separately funded and administratively managed in a ‘silo’) with the general health care system; multi-sectoral collaboration; or a combination of two or more of these meanings [10]. The World Health Organization (WHO) defines “*integrated health care” as “the organisation and management of health services so that people get the care they need, when they need it, in ways that are user-friendly, achieve the desired results and provide value for money*.” [10]. In this study, the ICDM model refers to the ‘one-stop-shop’ for the management of chronic diseases in PHC facilities as well as continuity of care in the form of referral of patients.

### Theoretical framework for evaluating quality of care in the ICDM model

“Quality of medical care” is highly contextual and a difficult concept to define. Although it is a reflection of values and goals in the medical care system and in the larger society which it is a part of, quality can be almost anything anyone wishes it to be [11]. Klein et al. conclude that patient care, like morale, cannot be defined by a unitary concept and that it seems unlikely that there will be a single criterion by which to measure the quality of patient care [12].

Avedis Donabedian described seven elements of quality of medical care: Efficacy, Effectiveness, Efficiency, Equity, Optimality, Acceptability and Legitimacy. Although *Efficacy* is hard to measure, it refers to care provided under optimal conditions and is the basis against which measurements should be made. *Effectiveness* describes the outcome of interventions; *Efficiency* refers to cost reductions without compromising effects; *Equity* refers to the fairness in the distribution of healthcare in populations; *Optimality* is about balancing the costs and benefits of healthcare; *Acceptability* encompasses accessibility of healthcare and interpersonal patient-provider interaction; and *Legitimacy* refers to the social acceptability of the healthcare institution regarding the manner in which healthcare is delivered. The choice of which of these elements, as well as their relative prioritisation, should be guided by the contexts in which quality of care is being assessed [13].

Donabedian’s definition of quality of care can be assessed as a triad of structure, process and outcome (SPO) constructs. He postulated that there are relationships between SPO constructs based on the idea that good structure should promote good process and good process should in turn promote good outcome (unidirectional pathway). The SPO framework, often represented by a chain of three boxes containing SPO constructs connected by arrows [13], can be used to draw inferences about the quality of health care [14]. Donabedian defines *Structure* as the professional and organisational resources associated with the provision of health care (e.g. availability of medicines/equipment and staff training); *Process* as the things done to and for the patient (e.g. defaulter tracing and hospital referrals) and *Outcome* as the desired result of care provided by the health practitioner (e.g. patient satisfaction with quality of care). Donabedian distinguished between two types of outcomes: i) technical outcomes, which are the physical and functional aspects of care, such as absence of complications and reduction in disease, disability and death; and ii) interpersonal outcomes which include patients’ satisfaction with care and influence of care on patient’s quality of life as perceived by the patient [15].

Avedis Donabedian’s SPO framework was used to evaluate the quality of care in the ICDM model not only because it is the dominant framework for evaluating the quality of medical care [16], but because the SPO framework is used by South Africa’s National Department of Health for implementing the ICDM model [9]. A study of quality systems conducted among department managers and quality coordinators in 386 hospitals in Sweden showed statistically significant relationships between SPO constructs, using Donabedian’s theory [17]. To the authors’ knowledge, this is the first study to apply Donabedian’s theory in evaluating the quality of care in the ICDM model in sub-Saharan Africa (SSA).

A systematic review to examine the effectiveness of integrating primary health services in Low- and Middle-Income Countries (LMICs) showed the main focus to be on the provider side of service provision, with virtually no considerations for lay or demand side perspective [18]. For South Africa, little is known about satisfaction with the quality of care in the ICDM model. With supporting evidence that satisfaction is a major component and key determinant of quality of healthcare [15], this study examined satisfaction of both service providers and users with the quality of care in the ICDM model. The objectives of this study were to: i) assess patients’ and operational managers’ (nurses-in-charge of health facilities) satisfaction with the dimensions of ICDM services; and ii) evaluate the quality of care in the ICDM model, based on the satisfaction scores of patients, using Donabedian’s SPO theoretical framework.

## Methods

### Study setting and sites

This study was conducted in PHC facilities in the rural Agincourt sub-district situated in the Bushbuckridge municipality, Mpumalanga province, northeast South Africa. At the time this study was conducted, the ICDM model was being implemented in 17 of the 38 PHC facilities in the sub-district. Seven of these 17 health facilities implementing the ICDM model are situated in Agincourt sub-district which covers an area of about 420 km^2^. The sub-district underpinned by a robust Health and Demographic Surveillance System (HDSS) which has been monitoring the population in these villages for two decades. The population under surveillance in the HDSS as at 1st July 2011 was 115,000 people in 20,000 households in 27 villages [19]. Three referral hospitals are situated 25 km to 45 km from the study setting. The pilot of the ICDM model was commenced in these facilities in June 2011 (field diary of interviews with the operational managers and the sub-district health manager in July 2013), but preceded by two months of pre-implementation preparedness which started in April 2011 [9]. Tsonga is the most widely spoken language in the study area. Having immigrated into South Africa mainly as war refugees in the early- and mid-1980s, one-third of the population in the study site are Mozambicans [19].

In the South African PHC model, the professional nurse is the service provider at the PHC facilities, which is the first point of entry into the public health system. Services provided by the nurses include: maternal and child care, immunization, family planning, treatment of sexually transmitted infections, minor trauma, care for chronic diseases and referrals. Medical doctors visit the PHC facilities at intervals to offer support to the nurses [20].

### Study design and study population

This was a cross-sectional survey conducted between August and November 2013. It was part of a broader four-year longitudinal study (January 2011 and December 2014), with qualitative and quantitative components, designed to contribute to understanding the effectiveness of the ICDM model in improving the quality of healthcare and technical health outcomes of chronic disease patients. The study population consisted of patients 18 years and above receiving treatment for chronic diseases in the sub-district health facilities. Other study participants included the operational managers (professional nurses-in-charge) of the selected seven PHC facilities in the sub-district.

### Inclusion and exclusion criteria for the patients

The ICDM model addresses the following chronic diseases: HIV/AIDS, tuberculosis, hypertension, diabetes, chronic obstructive pulmonary disease, asthma, epilepsy and mental health illnesses that are to be managed at the PHC level [9]. Considering the burden of chronic diseases in the study area, patients with markers of chronic diseases for HIV, hypertension and diabetes in the health facilities were included in the study, while those with other chronic diseases were excluded. Patients who had their chronic condition(s) managed five months before the initiation of the ICDM model until the time the study commenced in August 2013 were identified for recruitment. The reason for including patients receiving treatment five months before the ICDM model was implemented was to assess the levels of satisfaction of patients who had received treatment before the implementation of the ICDM model and continued to receive treatment during its implementation in efforts to gauge possible changes in the quality of chronic disease care attributable to the ICDM model. Minors less than 18 years were excluded from the study because they were below the age of autonomy (≥18 years) for judging satisfaction with the quality of services provided in the health facilities. The elderly with reduced capacity for comprehension during informed consent were also excluded from the study. Diminished capacity for comprehension was determined by the inability of prospective patients to comprehend or respond to the information verbally provided by the interviewer during informed consent.

### Sample size determination and sampling of study participants

Using the subjects-to-variables ratio (minimum of 10 subjects per variable in the study instrument) for estimating sample size for studies utilising factor analysis [21, 22], a sample size of 390 patient respondents was calculated (17 subjects per each of the 23 variables in the study instrument). The minimum sample size of approximately 435 (390/0.9) patients was reached after adjusting for 10% non-response. All the seven operational managers of the PHC facilities, the maximum number possible, were selected because they offered clinical services to the patients and the authors perceived their role as managers of the health facilities critically important to understanding the quality of the ICDM model more than other professional nurses.

The study participants were identified through a three-step process (Additional file 1). First, the number of patients recruited at each of the seven health facilities was determined by proportionate sampling. The sampling fraction of 435/3602 (435 represents the desired sample size out of a total of 3602 HIV, hypertension, and diabetes registered patients) was multiplied by the number of these chronic disease patients in each health facility to determine the number of patients to be recruited per facility. Secondly, the patients in each health facility were stratified by HIV, hypertension, and diabetes status in order to get a representative sample of the patients with markers of chronic diseases using a health facility-specific sampling frame. Finally, the numbers of patients specified in step two were recruited for a daily interview until the desired sample size in each clinic was achieved.

### Study tool and variables

In this study, we used the multi-scale patient satisfaction questionnaire (PSQ-18) which was developed by Ware et al. [23]. The PSQ-18 comprises 18 items derived from the full-length version (50-item) PSQ-III counterpart [23]. The PSQ-18 assesses multiple dimensions of patient satisfaction and includes general satisfaction; technical quality; interpersonal relations; communication; financial aspect; time spent with health provider; and accessibility and convenience (Additional file 2). The PSQ-18 sub-scales show acceptable reliability and correlate with the sub-scales in the PSQ-III [24]. Furthermore, PSQ-18 is appropriate for use in situations where there is need for brevity [24], as was the case in this study where it was administered to patients leaving the health facility after consultations with the nurses (patient exit interviews). The PSQ-18 instrument is reflective of Donabedian’s SPO constructs and succinctly measures patient satisfaction with dimensions of care for which SPO constructs are intended. The authors are not aware of any study that has used the PSQ-18 as a study instrument to operationalise Avedis Donabedian’s SPO theoretical framework in SSA.

Mahomed et al. described the innovative approaches in the HIV programme leveraged for NCDs by the NDoH [25]. From these, the study team consulted with the health facility managers and officers of the Mpumalanga Province Department of Health in selecting eight dimensions of care that patients are able to respond to as a result of their lived experiences with healthcare services in the PHC health facilities. The rationale for this selection was because some aspects of these innovative approaches were functions performed by nurses, laboratory staff and health policy implementers which patients were not privy to.

This study compared self-reported satisfaction of the patients and self-reported satisfaction of the operational managers with the dimensions of care listed in the ICDM model using the multi-scale PSQ-18. This is in view of literature depicting views of health care providers differing from users regarding the quality of health care [26]. Responses to statements were scored on a five-point Likert scale ranging from 4 (strongly agree) to 0 (strongly disagree) for positively-phrased statements, and from 4 (strongly disagree) to 0 (strongly agree) for negatively-phrased statements for the purpose of undertaking confirmatory factor analysis and structural equation modeling.

Similar to another study in which the PSQ tool was adapted to measure patient satisfaction with pharmacy services [27], this study adapted the PSQ-18 by altering a number of statements to fit the ICDM model. For example, the structure-related statement, “*I have easy access to the medical specialists I need*,” was changed to the ICDM-process-related dimension, “*Health care providers usually refer me to the doctor/hospital when there is need for the doctor to review me* - P5” (Additional files 2, 3 and 4). One structure-related (supply of critical medicines) and two process-related (defaulter tracing of patients and prepacking of medicines) variables were included in the adapted questionnaire. One process-related statement in the PSQ-18 was changed from “*health care providers act too business-like and impersonal toward me*” to “*Health care providers are professional in the conduct of their clinical duties*”. Regarding the types of outcome constructs (technical and interpersonal) specified by Donabedian, the focus of this study was on the subjective interpersonal outcome. Two outcome statements on “*satisfaction with perfect health care*” and “*dissatisfaction with some care*” in the PSQ-18 were changed to the dimension on “*satisfaction with coherent integrated chronic disease care*” and “*dissatisfaction with coherent integrated chronic disease care*”, respectively.

**Fig. 1:**
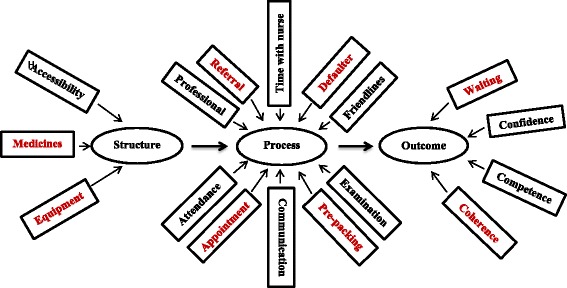
The 17 dimensions of care for which the structure, process and outcome constructs were intended. *The dimensions in red colour indicate the priority areas in the ICDM model

Two statements around the financial costs of health care (D1 and D2) were dropped during the adaptation of the PSQ-18 (Additional file 3). This is because the government of the Republic of South Africa implements a pro-equity policy, a component of free health care for everyone using the public primary health system [28]. However, transport-related costs were not considered in this study because it is not the responsibility of South Africa’ Department of Health to provide transport for the implementation of the ICDM model. The 17 dimensions of care in the adapted questionnaire are shown in Fig. 1, and details of the adapted PSQ tool used in the current study for patients and operational managers are shown in Additional files 3 and 4, respectively.

Eight dimensions of care were identified by experts on quality of care in the study team as priority areas for enhancing service efficiency and quality of care: supply of critical medicines, equipment, hospital referral, defaulter tracing, prepacking of medicines, clinic appointments, patient waiting time, and coherence of integrated chronic disease care (Additional files 5 and 6) [9]. This is because these priority areas are components of the tools and systems used in the successful HIV programme which is being leveraged to support or scale-up services for improving the quality of care for NCDs and patients interfaced directly with these areas in the health facilities (Fig. 1).

### Quality assurance

The adapted PSQ tool for the patients was forward translated to Tsonga (the local language) and back-translated to English by two experienced field workers who were blinded to each other. An experienced quantitative field worker was trained on how to administer the adapted PSQ tool. A pilot study was conducted in Cork clinic, a PHC facility situated outside the study site, to assure understanding and correct use of the PSQ tool. Only a few statements had to be rephrased after the pilot study.

An important characteristic of the original PSQ-18, which was considered in the adaptation of the study instrument, is the control for Acquiescent Response Set (ARS) - a tendency to agree with statements of opinion regardless of their content [29]. Acquiescent response set is a measurement error, specifically information bias, inherent in surveys assessing satisfaction with medical care. According to Ware et al. [29], there is a need to minimise information bias by assessing ARS in satisfaction surveys. Six variables were phrased in opposite directions, bringing to 23 the total number of variables in the adapted questionnaire (Additional files 3 and 4). These measures are beneficial in detecting skewness toward satisfaction [29] and identifying specific programme areas that respondents are satisfied or dissatisfied with.

### Data collection

Having consulted with the professional nurses and received their medicines, the prospective study participants were invited to a (consultation) room designated for patient interviews. Only the interviewer had access to this consultation room. Patients were invited to take part in the satisfaction survey after explaining the purpose of the study. They were assured that there will be no penalty or loss of benefits to which they were entitled to if they chose to not participate in this study or decide to discontinue participation in this study. Written informed consent was obtained from the patients who were willing to participate in the study and interviews were conducted with the patients.

### The operationalisation of Donabedian’s theoretical framework

The adapted PSQ contained measures reflective of SPO constructs and was used to assess satisfaction of patients and operational managers with the dimensions of integrated chronic disease services. There was no clear division of the statements in the adapted PSQ tool into the respective constructs. However, these statements have been categorised under these constructs in Additional files 3 and 4 for clarification. In order to minimise bias that may result from assessing acquiescent response set, the positive and negative statements did not follow each other in the questionnaire as shown in Additional files 3 and 4. The respondents were judged to be satisfied with the dimensions of care if the total relative frequency was ≥ 50% for “strongly agree” and “agree” responses to positively-phrased statements. Similarly, the respondents were judged to be satisfied with the dimensions of quality of care if the total relative frequency was ≥ 50% for “strongly disagree” and “disagree” responses to negatively-phrased statements. A satisfaction score of at least 50% was considered an average score using a scale of 0% to 100%.

The patients and operational managers were scored comparatively on their (dis)satisfaction with the dimensions of care in the ICDM model to measure the first objective of the study. Determining the quality of care in the ICDM model was the second objective of this study which was measured by conducting structural equation modelling (SEM) using the data on patients’ (dis)satisfaction with the dimensions of quality of care in the ICDM model. However, SEM could not be performed with the data collected from the operational managers because of the very small sample size (seven operational managers).

**Fig. 2:**
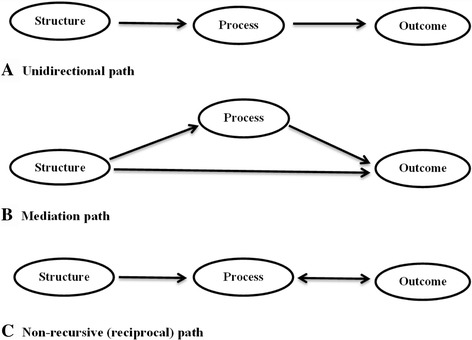
Pathways for operationalising Donabedian’s theory in the ICDM model of care in South Africa. **a** Unidirectional path: Good structure should promote good process and good process in turn should promote good outcome. **b** Mediation path: Good structure directly promotes good outcome, good structure promotes good process and good process in turn promotes good outcome. **c** Non-recursive (reciprocal) path: Good stucture promotes good process, good process promotes good outcome and good outcome in turn promotes good process

The following linear pathways were specified in the SEM: (1) the unidirectional pathway which states that good structure promotes good process and good process in turn promotes good outcome, (2) the mediation pathway which posits states that good structure directly promotes good outcome, good structure promotes good process and good process in turn promotes good outcome; and (3) the reciprocal pathway which hypothesises that good structure promotes good process, good process promotes good outcome and good outcome in turn promotes good process. The last two pathways were examined in this study to explore other linear relationships between SPO constructs other than the unidirectional pathway originally postulated by Donabedian (Fig. 2).

Fitting of the proposed pathways involved a four-step systematic process using patient data. First, a priori identification of the variables for which the SPO constructs were intended was performed by the experts on quality of care on the study team in order to assess the validity of the adapted questionnaire (Additional files 3 and 4). This method was adopted by Kunkel et al. in which a panel of experts categorised variables in a questionnaire into SPO constructs [17].

Secondly, Cronbach’s alpha (range: 0–1), which is a measure of internal consistency, was used to quantify the reliability of the multi-item variables in the adapted PSQ in measuring the SPO constructs. Cronbach’s alpha coefficient of reliability was categorised as excellent (α ≥ 0.9), good (0.7 ≤ α < 0.9), acceptable (0.6 ≤ α < 0.7), poor (0.5 ≤ α < 0.6) and unacceptable (α < 0.5) [30].

Next, the negative statements in the pair of statements phrased in opposite directions were dropped if there was no evidence of ARS. The fit of each construct and its individual items were assessed to remove any of the remaining variables with low coefficient of determination (CD < 0.2). Variables with low CD contribute high levels of error in the structural equation modelling [31]. Thereafter, Confirmatory Factor Analysis (CFA) was conducted to identify and remove the variables that did not load significantly (factor loading < 0.300) onto their intended constructs.

The following step used structural equation modelling (SEM) to assess the specified pathways, as used elsewhere [32], in order to determine the relationships between the SPO constructs (Fig. 2). Selection of the final path model was based on the variables that reflected their intended factors (factor loading ≥ 0.300). The Maximum Likelihood for Missing Values (MLMV) technique was used to impute for S5, P1 and P11 variables with 0.5%, 0.25% and 0.25% missing observations, respectively. The MLMV is a technique that handles missing data by estimating a set of parameters that maximise the probability of getting the data that was observed. It is a more superior and preferable method for handling missing data than the more popular multiple imputation [33], which is a simulation-based method that predicts missing values as close as possible to the true ones by replacing missing data with probable values based on other available information [34].

Assessment of the fit of the pathways using MLMV approach was based on two or more of the following fit indices [35]: (i) Relative/normed Chi-squared test statistic is an absolute fit index that assesses the discrepancy between observed and expected covariance matrices. It minimizes the impact of sample size on the model and is derived by dividing the Chi square value by the degrees of freedom (*χ* ^2^/df). Although there is no concensus regarding the acceptable ratio for this statistic, values ranging from 2 to 5 are recommended as good fit indices. [31]; (ii) Root Mean Squared Error of Approximation (RMSEA) is another absolute fit index that measures how well a model with optimally chosen parameter estimates fit the population’s covariance matrix - RMSEA value ≤ 0.06 is a good fit; (iii) Comparative Fit Index (CFI) is an incremental fit index that assesses the improvement in fit of the hypothesised model compared with a baseline (null) model, when population covariance is assumed to be zero - (CFI ≥ 0.90 is a good fit); (iv) Tucker-Lewis Index (TLI) is also an incremental fit index that corrects for model complexity by favouring parsimonious models over more complex ones - (TLI ≥ 0.90 is a good fit); and (v) Coefficient of determination (CD) indicates how well data fit a statistical model. We used CD to decide the model that explained the most variability. CD value of 1.00 is a perfect fit. The higher the number of criteria used, the better the fit of the model with the data [31].

### Statistical analysis

Data were entered into Access 2010 and imported into Stata 12.0 (College Station, TX, USA) for statistical analysis. Relative frequencies were used to quantify satisfaction of the patient and operational managers with the dimensions of integrated chronic disease services. At *p*-value ≤ 0.05, CFA and SEM were used to fit the specified structural path models in order to determine the quality of care in the ICDM model from the patient perspective.

## Results

### Socio-demographic characteristics of the patients

Table 1 shows the mean age of the 435 chronic disease patients to be 55 ± 16 years. Forty-eight percent of the patients were hypertensive; 81% females; 96% South Africans; 99% unemployed; and 90% were not looking for a paid job. Most of the patients received an old age grant (69%) and 88% of them had no formal or less than six years of education. The response rate for the patient interviews was 97%”

**Table 1:** Socio-demographic characteristics of the patients attending health facilities in Agincourt sub-district in 2013 (*n* = 435)

Variable	Frequency (%)
Age (years)	
18–29	23 (5.3)
30–39	69 (15.8)
40–49	68 (15.6)
50–59	88 (20.3)
60–79	187 (43.0)
Mean ± SD (55 ± 16.5); Median = 56	
Gender	
Female	354 (81.4)
Male	81 (18.6)
Education (years)	
No formal education	164 (37.6)
≤ 6	217 (49.9)
> 6	54 (12.5)
Type of grant	
None	91 (20.9)
Old age^a^	299 (68.7)
Disability	44 (10.1)
HIV	1 (0.3)
Labour status	
Not presently working	431 (99.0)
Presently working	4 (1.0)
Nationality	
South African	415 (95.5)
Mozambican	20 (4.5)
Chronic disease status^b^	
Hypertension	292 (67.0)
HIV	141 (32.4)
Diabetes	2 (0.5)

^a^Old age grant is a social security grant given to South Africans ≥ 60 years of age

^b^Diagnoses of chronic diseases were retrieved from the patients’ clinic records

### Satisfaction with structure-, process- and outcome-related dimensions of care in the ICDM model

**Fig. 3:**
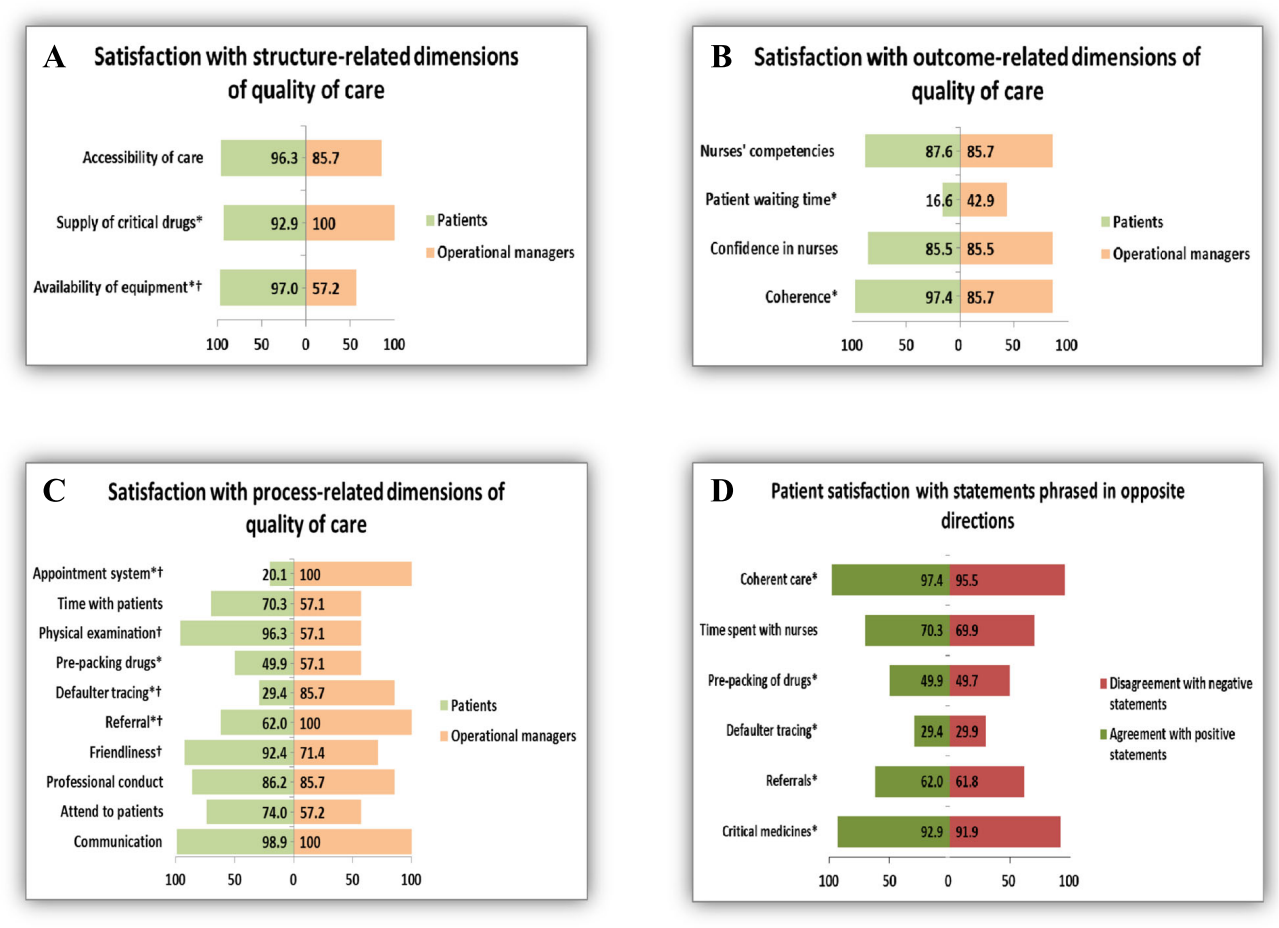
Satisfaction of respondents with the ICDM model and assessment of acquiescent response set for patients. *Priority areas in the ICDM model ^†^ *p*-value < 0.05. **a** Satisfaction with structure-related dimensions of quality of care. **b** Satisfaction with process-related dimensions of quality of care. **c** Satisfaction with outcome-related dimensions of quality of care. **d** Patient satisfaction with statements phrased in opposite directions

Figure 3a shows that the patients (P) and operational managers (OM) reported being satisfied (scores ≥ 50%) with all the structure-related dimensions of care in the ICDM model. There were no statistically significant differences (*p* > 0.05) between the satisfaction scores of the patients and operational managers with structure-related dimensions of care, except for availability of equipment (S1): P-97% vs. OM-52%, *p* < 0.001.

Figure 3b shows that the operational managers reported being satisfied (scores ≥ 50%) with all process-related dimensions of care in the ICDM model. However, the patients were not satisfied (scores < 50%) with defaulter tracing of patients (P7-29%) and appointment systems (P14-20%). Of all the process-related dimensions of care, there were statistically significant differences in the scores of the patients and operational managers in appointment system (P14): P-20% vs. OM-100%, *p* < 0.001; physical examination of patients (P11): P-96% vs. OM-57%, *p* < 0.001; defaulter tracing of patient (P7): P-29% vs. OM-86%, *p* = 0.001; hospital referral of patients (P5): P-62% vs. OM-100%, *p* = 0.039; and friendliness of the nurses to patients (P4): P-92% vs. OM-71%, *p* = 0.041; .

Figure 3c shows that the patients and operational managers reported being satisfied (scores ≥ 50%) with three of the four outcome-related dimensions of care in the ICDM model. On the other hand, the patients and operational managers were not satisfied (scores < 50%) with patient waiting time (O4): P-17% vs. OMs-43%. A comparison of the satisfaction scores of the patients and operational managers with all the outcome-related dimensions of care showed no statistically significant differences (*p* > 0.05).

### Acquiescent response set

Figure 3d shows patients’ satisfaction scores for the positively- and negatively-phrased statements: supply of critical drugs (93% vs. 92%), hospital referrals (62% vs. 62%), defaulter tracing (29% vs. 30%), prepacking of drugs before clinic visits (50% vs. 50%), time nurses spent with patients during consultation (70% vs. 70%) and coherence of integrated chronic disease care (97% vs. 96%). There were no statistically significant differences (*p* > 0.05) in the responses of the patients to the pair of positively- and negatively-phrased statements.

### Fitting of the proposed structural pathways

**Fig. 4:**
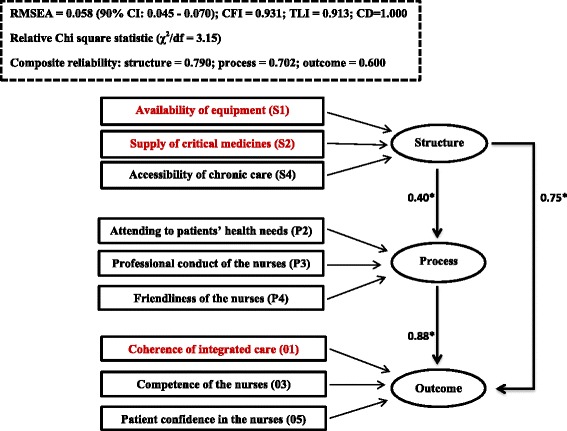
Goodness-of-fit, reliability and correlation assessment of the relationships between structure, process and outcome. *Relationships between the constructs represented by the Pearson correlation values. NB: The dimensions in red colour are the priority areas in the ICDM model. RMSEA - Root Mean Squared Error of Approximation (≤0.06 is a good fit). CFI - Comparative Fit Index (CFI ≥ 0.90 is a good fit). TLI - Tucker-Lewis Index (TLI ≥ 0.90 is a good fit). CD - Coefficient of determination (range 0–1. There is a perfect fit of the data with the model if CD = 1). Cronbach’s alpha coefficient of reliability (≥0.6 is acceptable)

Figure 4 shows that the Cronbach’s alpha coefficients of reliability of the variables intended for their respective SPO constructs ranged from acceptable to good: structure (0.790), process (0.702), and outcome (0.600), an indication that the variables were a reliable measure of their intended constructs [30].

Before running the factor analysis, six negatively phrased statements (S3, P6, P8, P10, P13 and O2) in the adapted questionnaire were dropped because there was no evidence of ARS in the pair of statements phrased in opposite directions. In assessing the fit of the constructs and the remaining 17 variables, three process-related variables: communication with patients (P1), hospital referral (P5) and physical examination of patients (P11) with coefficient of determination values < 0.20 were dropped [31]. Of the remaining 14 variables, four process-related variables: defaulter tracing of patients (P7), prepacking of drugs before clinic visit (P9), time patients spent with nurses during consultation (P12) and appointment system (P14); and one outcome-related variable: patient waiting time (O4) did not load significantly (factor loadings < 0.3) onto their intended constructs in the CFA (Table 2), and were dropped after CFA.

**Table 2:** The result of the confirmatory factor analysis

Constructs	Variables	Loading	Standard error
Structure			
	Availability of equipment (S1)	0.462^a^	0.038
	Supply of critical medicines (S2)	0.994^a^	0.012
	Accessibility of services (S4)	0.383^a^	0.041
Process			
	Attendance to patients’ needs (P2)	0.664^a^	0.035
	Professionalism (P3)	0.758^a^	0.032
	Friendliness (P4)	0.669^a^	0.035
	Defaulter tracing (P7)	0.200	0.056
	Prepacking of drugs (P9)	0.268	0.055
	Time spent with nurses (P12)	0.074	0.056
	Appointment system (P14)	0.163	0.053
Outcome			
	Coherence (O1)	0.310^a^	0.057
	Competence (O3)	0.485^a^	0.053
	Waiting time (O4)	0.229	0.058
	Confidence (O5)	0.651^a^	0.054

^a^Variables with factor loading ≥ 0.300

### Assessment of fit indices of the specified path models

Figure 4 also shows the remaining nine variables that reflected their intended SPO constructs (factor loading > 0.300) in the structural equation model. These were three structure-related dimensions: availability of equipment (S1), supply of critical medicines (S2) and accessibility of chronic disease care (S4); three process-related dimensions: attending to patients’ health needs (P2), professional conduct of the nurses (P3) and friendliness of the nurses (P4); and three outcome-related dimensions: coherence of integrated chronic disease care (O1), patient confidence in the nurses (O3), and competence of the nurses (O5).

The fit indices of the three specified pathways are as follows: (a) unidirectional pathway – [*χ* ^2^/df = 2.44; RMSEA = 0.064 (90% CI - 0.052–0.077); CFI = 0.915; TLI = 0.892; CD = 0.911]; (b) mediation pathway – [*χ* ^2^/df = 3.15; RMSEA = 0.058 (90% CI - 0.045–0.070); CFI = 0.931; TLI = 0.913; CD = 1.00; and (c) reciprocal pathway – [*χ* ^2^/df = 2.78; RMSEA = 0.059 (90% CI - 0.047–0.070); CFI = 0.919; TLI = 0.910; CD = 0.632].

Table 3 showed that when using at least two criteria, all the specified path models fit the data, but only the mediation pathway fulfilled all the criteria used.

**Table 3:** The result of the goodness of fit of the specified path models

Criteria	Specified path models		
	Unidirectional	Mediation	Reciprocal
Relative Chi square statistic (*χ* ^2^/df)	127/52 = 2.44✓	164/52 = 3.15✓	145/52 = 2.78✓
RMSEA value ≤ 0.06	0.064 (90% CI - 0.052–0.077)	0.058 ✓ (90% CI - 0.045–0.070)	0.059 ✓ (90% CI - 0.047–0.070)
CFI ≥ 0.90	0.915 ✓	0.931 ✓	0.919 ✓
TLI ≥ 0.90	0.892	0.913 ✓	0.910 ✓
CD close to 1.00 (perfect fit is preferred if CD value = 1.00)	0.911 ✓	1.00 ✓	0.632
Ranking**	3^rd^	1^st^	2^nd^

✓Indices with goodness of fit

**The mediation model ranked first because it fulfilled all five criteria (Relative/normed Chi square statistic, RMSEA, CFI, TLI and CD). In addition, it showed a perfect fit based on CD value of 1.00

**The reciprocal model ranked second because it fulfilled four criteria (Relative/normed Chi square statistic, RMSEA, CFI and TLI)

**The unidirectional model ranked third because it fulfilled three criteria (Relative/normed Chi square statistic, CFI and CD)

### Summary of the main findings

The patients and operational managers’ were satisfied (scores ≥ 50%) with the following SPO related dimensions of care:

i) structure-related construct: availability of equipment; supply of critical medicines; and accessibility of chronic disease care.
ii) process-related construct: communication of the nurses with patients; attendance of the nurses to patients’ health needs; professional conduct of the nurses; nurses’ friendliness with patients; hospital referral of patients, pre-packing of medicines; physical examination of patients; and time nurses spent with patients during consultation
iii) outcome-related: coherence of integrated chronic disease care; and competence of the nurses, and patients’ confidence in the nurses.

The patients and operational managers’ were less satisfied (scores < 50%) with patient waiting time (an outcome construct). The patients recorded satisfaction scores < 50% for two process-related dimensions of care, defaulter tracing of patients and appointment systems. There were statistically significant differences (*p* < 0.05) in the satisfaction scores of the patients and operational managers with regard to availability of equipment; friendliness of the nurses; hospital referral of patients; defaulter tracing of patients; physical examination of patients; and appointment systems.

Findings from the mediation path model (Fig. 4) showed that three structure-related dimensions of care (availability of equipment; supply of critical medicines; and accessibility of chronic disease care) correlated directly with three outcome-related dimensions of care (coherence of integrated chronic disease care; and competence of the nurses and patient confidence in the nurses) and three process-related dimensions of care (nurses’ friendliness with patients; professional conduct of the nurses; and attendance of the nurses to patients’ health needs). Independent of structure, good process correlated with good outcome, an indication that good process mediated the relationship between good structure and good outcome.

## Discussion

In view of the increasing emphasis on health system strengthening and integration, this study contributes to the national and global debates on the feasibility of integrating HIV services with those of NCDs. More specifically, we examined the satisfaction of patients and operational managers with the dimensions of integrated chronic disease services and evaluated the quality of care in the ICDM model from patient perspectives using Donabedian’s theory of the relationships between SPO constructs as a measure of the quality of care.

Similar to a Togolese study in which the majority of service providers positively viewed the impact of integrating family planning services to the routine expanded programme on immunisation [36], the operational managers in this study reported being satisfied with 16 of the 17 dimensions of quality of care in the ICDM model. However, this was less so for the patients who reported satisfaction with 14 of these dimensions of care. The significant differences in the satisfaction scores of the patients and operational managers in this study supports evidence-based literature that suggests assessing the satisfaction of the quality of care from the perspectives of both health providers and users [18] because of differing views [26]. The patients rated satisfaction with availability of equipment higher than the operational managers because the patients may not be aware of the lack of equipment. The patients’ satisfaction scores for friendliness of the nurses and physical examination of patients was higher than those of the operational managers. The operational managers who responded to the interviews were professional nurses who often performed a dual role of providing routine care to the patients and managing the facilities. In the course of performing their administrative duties in the office, these managers may not have the opportunity to see other professional nurses being friendly to patients in the consultation rooms. This may have accounted for the managers’ lower satisfaction scores compared with the patients’ scores.

An earlier household survey conducted in the study site reported health system weakness as one of the barriers to chronic disease care. At the time of the survey in 2004, community members attended public hospitals for diagnosis and treatment of chronic illness due to the lack of capacity and services in the PHC facilities [37]. A decade after the 2004 survey and two years after the initiation of the ICDM model in South Africa, community members now have access to chronic disease services in PHC facilities in their local areas. These facilities have a more regular supply of critical drugs and trained professional nurses who are better able to provide integrated services for the diagnosis and treatment of chronic diseases. This may be an indication some progress that has been made in chronic disease care in the study setting.

In this study, patient waiting time was the only dimension of care in the ICDM model in which the patients and operational managers reported low satisfaction scores. Similar studies assessing the quality of service in public clinics in South Africa showed that the clinics were easily accessible and services were of acceptable quality [38], but the time spent by patients at the clinic to complete the services was very long [38, 39]. These findings suggest that public health services in many resource-constrained LMICs are characterised by long waiting periods [40–42], which could be a consequence of operational challenges such as performance of multiple tasks and work overload of health workers [18]. In addition to staff shortage which was reported by operational managers and patients in the qualitative component [43] of the broader mixed methods study, patients who missed previous clinic appointments were being made to wait in the queues during subsequent visits until nurses had attended to patients who were on the appointment list for that day [43]. Other factors reported by patients as contributing to long waiting time in the qualitative study were late arrival of filing clerks and nurses; long morning prayer sessions before commencement of clinical duties; staff meetings; prolonged tea or lunch breaks; and nurses giving preferential treatment to friends or relatives who skip the queues [43].

The lack of an Acquiescent Response Set (ARS) found in this study does not support literature evidence that suggests patient satisfaction surveys are almost always skewed toward satisfaction with positively worded statements [23]. The reasonable explanation for the absence of ARS in this study can be attributed to two factors: (1) the fieldworker received training on how to read the statements in the interviewer-administered questionnaire very slowly and carefully to the patients in a way that the statements were understood, and (2) the questionnaire was pre-tested to provide feedback to the study team. The purpose of testing for ARS in this study was to minimise information bias [29] by checking to see if the patients understood the statements in the adapted PSQ-18. As implementation of ARS does not eliminate coercion, we addressed the possibility of coercion, which is more likely to occur in people of low socioeconomic status by assuring patients that there would no penalty or loss of patient benefits if they chose not to participate or decided to discontinue participation at any point in the study.

A Swedish study used Donabedian SPO theoretical framework to show a statistically significant relationship between SPO constructs through the mediation pathway [17]. This research corroborates the Swedish study and further reinforces the usefulness of Donabedian’s theory in evaluating the quality of healthcare generally, and more specifically in the context of the ICDM model. The perception of the patients about the quality of care in the ICDM model can be interpreted to mean that the provision of good structure directly promotes good outcome; and that the relationship between good structure and good outcome is mediated by good process. More specifically, the patients thought that the provision of equipment, drugs and accessibility of chronic disease services contributed to the nurses’ ability to be professional in their duties, become friendly to patients and attend to patients health needs. If the nurses performed these duties, the patients had confidence in the nurses, thought that the nurses were competent, and perceived there was coherence in the services provided by the nurses.

Although Donabedian’s framework continues to be the dominant touchstone paradigm for assessing the quality of health care, it has been described as too linear to recognise complex interactions between SPO constructs [16]. Donebedian’s critics argue that his theory fails to incorporate patient characteristics which are important precursors in the evaluation of the quality of health services [44]. However, these limitations do not affect the validity of our study for the following reasons. First, the linearity of Donabedian’s theory forms the basis of our study which assesses the linearity of the relationships between SPO constructs through the specified unidirectional, mediation and reciprocal pathways. The linearity of Donabedian’s theory would have been a limitation in our study if we sought to determine non-linear relationships between SPO constructs . Regarding the limitation of Donabedian’s theory not accounting for patients’ socio-demographic characteristics, it is important to note that patients’ characteristics could not have been categorised as dimensions of care under SPO constructs in the ICDM model. This is because patients’ characteristics do not fit into Donebedian’s definition of SPO constructs and therefore have no role to play in explaining Donabedian’s theory of quality of care; hence, the rationale for selecting his theoretical framework for evaluating the quality of care in the ICDM model.

### Implications

Of the eight priority areas in the ICDM model (supply of critical medicines, equipment, hospital referral, defaulter tracing, prepacking of medicines, clinic appointments, reducing patient waiting time, and coherence of integrated chronic disease care), the supply of critical medicines, availability of equipment and coherence of integrated chronic disease care reflected their intended constructs in the final model. On the other hand, the remaining five priority areas (hospital referral, defaulter tracing, prepacking of medicines, clinic appointments, and reducing patient waiting time) di not reflect their intended constructs.

The authors suggest an interaction of factors responsible for why hospital referral, defaulter tracing, prepacking of medicines, clinic appointments, and reducing patient waiting time did not reflect their intended constructs in the study settings. For instance, patient waiting time may have been unnecessarily prolonged in the study settings due to many factors. The purpose of prepacking medicines before patient arrival is to reduce patient waiting time; however, the high rate of patient’s missed appointments and unavailability of prepacking bags could have served as a deterrent from nurses prepacking medicines [43].

### Study strengths and limitations

The main strength of this study was the use of the patient satisfaction survey to evaluate the quality of care in the ICDM model in PHC facilities in a rural setting in South Africa using Donabedian’s theory. In addition, we assessed satisfaction with integrated chronic disease services, from the perspectives of healthcare providers and users. This study also provided insight on some of the priority areas of the ICDM model in the study settings. Study findings should be interpreted in light of the following limitations: (1) Causal relationships between SPO constructs cannot be inferred because this study was cross-sectional by design, (2) Data on interpersonal outcomes (dis)satisfaction with care do not necessarily reflect technical outcomes (e.g. reduced diseases, disabilities and deaths) in the ICDM model of care in the study settings, (3) The perspectives of clinic defaulters were not taken into account, (4) Inferences could not be made about the (dis)satisfaction of other professional nurses with services in the ICDM model, due to the small number of operational managers who were interviewed, and (5) It was not feasible to include all the priority dimensions of care in the questionnaire because patients were not privy to some of these aspects of care in the ICDM model.

## Conclusion

The patients and operational managers were satisfied with many areas of the integrated chronic disease services, but had divergent opinions about satisfaction with some dimensions of care. Of the 17 dimensions of care assessed in the ICDM model, nine refelected their intended constructs. Five of the eight priority areas assessed in the ICDM model (hospital referral, defaulter tracing, prepacking of medicines, clinic appointments, and patient waiting time) did not reflect their intended constructs; hence the need to strengthen services in these areas.

## Additional files

Additional file 1: Sampling of study participants. (PDF 95 kb)

Additional file 2: Patient satisfaction questionnaire-18 developed by Ware et al. (PDF 285 kb)

Additional file 3: Patient satisfaction questionnaire adapted for patients in the study. (PDF 90 kb)

Additional file 4: Patient satisfaction questionnaire adapted for operational managers in the study. (PDF 190 kb)

Additional file 5: Innovative approaches in the HIV programme leveraged for NCDs in the ICDM model by the NDoH in South Africa. (PDF 89 kb)

Additional file 6: Table of definition of terms used in the article. (PDF 97 kb)

## Abbreviations

ARS: Acquiescent response set
ART: Antiretroviral treatment
CD: Coefficient of determination
CFA: Confirmatory factor analysis
CFI: Comparative fit index
CHWs: Community health workers
HDSS: Health and demographic surveillance system
HIV/AIDS: Human immunodeficiency virus/acquired immune deficiency syndrome
ICDM: Integrated chronic disease management
LMICs: Low- and middle-income countries
MLMV: The maximum likelihood for missing values
NCDs: Non-communicable diseases
NDoH: National Department of Health
PHC: Primary health care
PSQ: Patient satisfaction questionnaire
RMSEA: Root mean squared error of approximation
SEM: Structural equation modelling
SPO: Structure, process, and outcome
SSA: Sub-Saharan Africa
TLI: Tucker-Lewis Index
UNAIDS: Joint United Nations Programme on HIV/AIDS
WHO: World Health Organization.
