# Supplementary material for: Relationships between structure, process and outcome to assess quality of integrated chronic disease management in a rural South African setting: applying a structural equation model
Source: BMC Health Serv Res. 2017 Mar 23;17:229. doi: 10.1186/s12913-017-2177-4 (PMC5363044; doi:10.1186/s12913-017-2177-4)
Supplement: Supplementary file 1 — Sampling of study participants. (PDF 95 kb) [file 12913_2017_2177_MOESM1_ESM.pdf]

Health facilities	Number of patients recorded in the clinic appointment roaster in July 2013.				***Step 1 sampling**: proportionate sampling from the health facilities	****Step 2 sampling**: stratified sampling by chronic disease status		
	ART	HPT	DM	Total		ART	HPT	DM
A	724	642	-	1366	165	88	77	0
B	146	715	-	861	104	18	86	0
C	41	325	7	373	45	5	39	1
D	84	274	6	364	44	10	33	1
E	66	50	-	116	14	8	6	0
F	50	215	-	265	32	6	26	0
G	49	208	-	257	31	6	25	0
Total	1160	2429	13	3602	435	141	292	2

#HIV, HPT and DM = HIV/AIDS, hypertension and diabetes mellitus patients, respectively.

***Step 1: proportionate sampling for each health facility was achieved by multiplying the sampling fraction by the total number of patients in each health facility**

Sampling fraction = 435/3602 = 0.1207

Where 435 = calculated study sample size and 3602 = total sampling frame

Example of proportionate sampling for clinic A: 0.1207 x 1366 = 165, where 1366 is the total number of patients in health facility A.

****Step 2: stratified sampling in each health facility**

Example of stratified sampling in health facility A

53% (724/1366) and 47% (642/1366) of the total number of patients in health facility A were HIV patients. Of the 165 patients recruited in health facility A, 53% (n=88) were HIV patients and 47% (n=77) were hypertension patients.
