# Supplementary material for: Relationships between structure, process and outcome to assess quality of integrated chronic disease management in a rural South African setting: applying a structural equation model
Source: BMC Health Serv Res. 2017 Mar 23;17:229. doi: 10.1186/s12913-017-2177-4 (PMC5363044; doi:10.1186/s12913-017-2177-4)
Supplement: Supplementary file 3 — Patient satisfaction questionnaire adapted for patients in the study. (PDF 90 kb) [file 12913_2017_2177_MOESM3_ESM.pdf]

Labels	Statements
Structure	
S1	I think my health care providers have functional equipment to provide complete chronic disease care
S2 +	I think this health facility has regular supply of medicines for the treatment of my illness[a]
S3 -	I think this health facility does not have regular supply of medicines for the treatment of my illness[a]
S4	I am able to get health care whenever I need it
Process	
P1	Health care providers are good about explaining the reason for medical tests
P2	Health care providers sometimes ignore what I tell them
P3	Health care providers are professional in the conduct of their clinical duties
P4	My health care providers treat me in a very friendly and courteous manner
P5 +	Health care providers usually refer me to the hospital when there is need for the doctor to review me[a]
P6 -	Health care providers do not refer me to the hospital when there is need for the doctor to review me[a]
P7 +	When I miss clinic appointment(s), the Community (volunteer) Health Workers visit my house to ask why I did not come to the clinic[a]
P8 -	The Community (volunteer) Health Workers do not visit my house when I miss clinic appointment(s)[a]
P9 +	Health care providers prepack my drugs the day before my clinic appointment(s)[a]
P10 -	Health care providers do not prepack my drugs the day before my clinic appointment(s)[a]
P11	When I visit the clinic, the providers are careful to check everything when treating and examining me
P12 +	Health care providers usually spend plenty of time with me
P13 -	Those who provide my health care sometimes hurry too much when they treat me
P14	I find it hard to get an appointment when I need it[b]
Outcome	
O1 +	I am satisfied with the quality of coherent integrated chronic disease care that I receive[b]
O2 -	I am dissatisfied with the quality of coherent integrated chronic disease care that I receive[b]
O3	Sometimes health care providers make me wonder if their diagnosis is correct
O4	When I come to receive health care, i have to wait for too long to access services[b]
O5	I have some doubts about the ability of the health care providers who treat me
Dropped	
D1 +	I feel confident that I can get the health care I need without being set back financially
D2 -	I have to pay for more of my health care than I can afford

[a]Priority areas of the ICDM model in South Africa
[b]Priority areas of the ICDM model in South Africa originally contained in the PSQ-18
+ sign indicates positive statements phrased in opposite directions
- sign indicates negative statements phrased in opposite directions
Dropped: statements excluded from the adapted PSQ because of free PHC service offered in South Africa
