# Supplementary material for: Relationships between structure, process and outcome to assess quality of integrated chronic disease management in a rural South African setting: applying a structural equation model
Source: BMC Health Serv Res. 2017 Mar 23;17:229. doi: 10.1186/s12913-017-2177-4 (PMC5363044; doi:10.1186/s12913-017-2177-4)
Supplement: Supplementary file 4 — Patient satisfaction questionnaire adapted for operational managers in the study. (PDF 190 kb) [file 12913_2017_2177_MOESM4_ESM.pdf]

| Labels | Statements |
|---|---|
| Structure | |
| S1 | I think the health care providers have functional equipment to provide complete chronic disease care |
| S2 + | I think this health facility has regular supply of medicines for the treatment of chronic diseases[a] |
| S3 - | I think this health facility does not have regular supply of medicines for the treatment of chronic diseases[a] |
| S4 | The patients are able to get health care whenever they need it |
| | |
| Process | |
| P1 | Health care providers are good about explaining the reason for medical tests |
| P2 | Health care providers sometimes ignore what patients tell them |
| P3 | Health care providers are professional in the conduct of their clinical duties |
| P4 | Health care providers treat chronic disease patients in a very friendly and courteous manner |
| P5 + | Health care providers usually refer patients to the hospital when there is need for the doctor to review them[a] |
| P6 - | Health care providers do not refer patients to the hospital when there is need for the doctor to review them[a] |
| P7 + | When patients miss clinic appointment(s), the Community (volunteer) Health Workers visit their houses to ask why they did not come to the clinic[a] |
| P8 - | The Community (volunteer) Health Workers do not visit patients' houses when they miss clinic appointment(s)[a] |
| P9 + | Health care providers prepack patients' drugs the day before their clinic appointment(s)[a] |
| P10 - | Health care providers do not prepack patients' drugs the day before their clinic appointment(s)[a] |
| P11 | When patients visit the clinic, the providers are careful to check everything when treating and examining them |
| P12 + | Health care providers usually spend plenty of time with patients |
| P13 - | Those who provide health care sometimes hurry too much when they treat patients |
| P14 | Patients find it hard to get an appointment when they need it[b] |
| | |
| Outcome | |
| O1 + | I am satisfied with the quality of coherent integrated chronic disease care patients receive[b] |
| O2 - | I am dissatisfied with the quality of coherent integrated disease care patients receive[b] |
| O3 | Sometimes health care providers make me wonder if their diagnosis is correct |
| O4 | When patients come to receive health care, they have to wait for too long to access services[b] |
| O5 | I have some doubts about the ability of the health care providers who treat the patients |

[a]Priority areas of the ICDM model in South Africa
[b]Priority areas of the ICDM model in South Africa originally contained in the PSQ-18
+ sign indicates positive statements phrased in opposite directions
- sign indicates negative statements phrased in opposite directions
