# Supplementary material for: Relationships between structure, process and outcome to assess quality of integrated chronic disease management in a rural South African setting: applying a structural equation model
Source: BMC Health Serv Res. 2017 Mar 23;17:229. doi: 10.1186/s12913-017-2177-4 (PMC5363044; doi:10.1186/s12913-017-2177-4)
Supplement: Supplementary file 5 — Innovative approaches in the HIV programme leveraged for NCDs in the ICDM model by the NDoH in South Africa. (PDF 89 kb) [file 12913_2017_2177_MOESM5_ESM.pdf]

Innovative approaches/priority dimensions of care	Categorisation of dimensions under structure, process and outcome constructs
**<u>Dimensions listed in the article published by Mahomed et al.</u>**	
Multidisciplinary teams	
Task-shifting	
**Appointment systems**	Process
Standardised medical records	
Infrastructure	
**Equipment**	Structure
Strengthening laboratory systems	
**Referrals**	Process
Improved supply chain management and procurement	
New cadres of health workers	
Adherence support*	
Patient education*	
Community outreach through home-based services **(defaulter-tracing)**	Process
Community engagement	
Stakeholder participation	
Improved monitoring and evaluation	
**<u>Dimensions listed in the ICDM manual (Mahomed is a co-author of this manual)</u>**	
**Critical medicines (page 62)**	Structure
**Waiting time (page 63)**	Outcome
**Coherence of integrated chronic care (page 78)**	Outcome
**Prepacking/pre-dispensing of medication (page 78)**	Process

# The eight emboldened dimensions of care were selected based on the ability of patients to respond to statements in the questionnaire based on their lived experiences with healthcare services in the health facilities

**Adherence support and patient education were not included in the questionnaire because services in these areas focused on HIV/AIDS patients who constituted 32% of the study population in our study*
