# Supplementary material for: Relationships between structure, process and outcome to assess quality of integrated chronic disease management in a rural South African setting: applying a structural equation model
Source: BMC Health Serv Res. 2017 Mar 23;17:229. doi: 10.1186/s12913-017-2177-4 (PMC5363044; doi:10.1186/s12913-017-2177-4)
Supplement: Supplementary file 6 — Table of definition of terms used in the article. (PDF 97 kb) [file 12913_2017_2177_MOESM6_ESM.pdf]

Terms	Definition
Critical medicines	Anti-hypertension, antiretroviral and anti-diabetic drugs
Hospital referral	Referral of chronic disease patients by the professional nurses to the district hospitals
Defaulter tracing	Search for patients who did not attend a clinic following previous clinic appointment
Prepacking of medicines	Packing of patients' medicines before commencement of routine clinic activities
Clinic appointments	An appointment system in which a healthcare worker books a patient for a subsequent follow-up visit
Patient waiting time	The period between the arrival of patients to a health facility and when they leave the health facility
Coherence of integrated chronic disease care	Consistency and continuity in the provision of care that is convenient for patients
